# DUX4c Is Up-Regulated in FSHD. It Induces the MYF5 Protein and Human Myoblast Proliferation

**DOI:** 10.1371/journal.pone.0007482

**Published:** 2009-10-15

**Authors:** Eugénie Ansseau, Dalila Laoudj-Chenivesse, Aline Marcowycz, Alexandra Tassin, Céline Vanderplanck, Sébastien Sauvage, Marietta Barro, Isabelle Mahieu, Axelle Leroy, India Leclercq, Véronique Mainfroid, Denise Figlewicz, Vincent Mouly, Gillian Butler-Browne, Alexandra Belayew, Frédérique Coppée

**Affiliations:** 1 Laboratory of Molecular Biology, University of Mons-Hainaut, 6, Mons, Belgium; 2 INSERM ERI 25 Muscle et Pathologies, CHU A. de Villeneuve, Montpellier, France; 3 Eppendorf Array Technologies, Namur, Belgium; 4 Department of Neurology, University of Michigan, Ann Arbor, Michigan, United States of America; 5 Institute of Myology, Platform for human cell culture, Paris, France; Katholieke Universiteit Leuven, Belgium

## Abstract

Facioscapulohumeral muscular dystrophy (FSHD) is a dominant disease linked to contractions of the *D4Z4* repeat array in 4q35. We have previously identified a double homeobox gene (*DUX4*) within each *D4Z4* unit that encodes a transcription factor expressed in FSHD but not control myoblasts. DUX4 and its target genes contribute to the global dysregulation of gene expression observed in FSHD. We have now characterized the homologous *DUX4c* gene mapped 42 kb centromeric of the D4Z4 repeat array. It encodes a 47-kDa protein with a double homeodomain identical to DUX4 but divergent in the carboxyl-terminal region. DUX4c was detected in primary myoblast extracts by Western blot with a specific antiserum, and was induced upon differentiation. The protein was increased about 2-fold in FSHD versus control myotubes but reached 2-10-fold induction in FSHD muscle biopsies. We have shown by Western blot and by a DNA-binding assay that DUX4c over-expression induced the MYF5 myogenic regulator and its DNA-binding activity. DUX4c might stabilize the MYF5 protein as we detected their interaction by co-immunoprecipitation. In keeping with the known role of Myf5 in myoblast accumulation during mouse muscle regeneration DUX4c over-expression activated proliferation of human primary myoblasts and inhibited their differentiation. Altogether, these results suggested that DUX4c could be involved in muscle regeneration and that changes in its expression could contribute to the FSHD pathology.

## Introduction

Facioscapulohumeral muscular dystrophy 1A (FSHD1A, OMIM #158900) is the third most frequent hereditary disease of muscle, affecting one individual in 20,000 and is associated with contractions of a repeat array in the subtelomeric 4q35 region [1]–[3]. In non-affected individuals the array comprises 11–100 tandem copies of a 3.3-kilobase (kb) element named *D4Z4*. In patients, only 1–10 *D4Z4* copies are left, and the disease is usually more severe with shorter repeat arrays [4]–[7]. It is chromosome-4 specific since contractions of the homologous repeat array in 10q26 do not cause FSHD [8]. Additional features are also needed on chromosome 4 besides the array contraction since neither the 4qB allele [9] nor some 4qA polymorphic alleles are linked to FSHD [10].

Several genes identified in the region proximal to *D4Z4* might contribute to the full FSHD phenotype (reviewed in [11]). Various mechanisms were proposed to explain how the deletion might activate their expression in FSHD muscles (reviewed in [12]; [13]). A FSHD-related nuclear matrix attachment site (FR-MAR) was recently mapped in the locus [13] that establishes a first chromatin loop containing the D4Z4 array and a second 150-kb loop containing *FRG1* (FSHD region gene 1) [14], [15], *TUBB4Q* (a tubulin pseudogene) [15] and *FRG2* (FSHD region gene 2) [16]. In addition a very potent transcriptional enhancer was found in the 5′-part of the D4Z4 unit ([17]). The FR-MAR has an enhancer blocking activity that is efficient in human control myoblasts and non-muscle cells. However in FSHD the FR-MAR is weakened so that the repeat array and its neighbouring genes are brought into a single chromatin loop where the D4Z4 enhancer might up-regulate transcription of any gene ([13], [17]) including the *DUX4* (double homeobox) gene that our group mapped inside each *D4Z4* element [18]. Among those genes, *FRG1* was recently shown to be involved in muscle development in Xenopus [19], and transgenic mice that strongly over-express FRG1 in their skeletal muscles present a muscular dystrophy, suggesting an involvement in the disease [20].

Our group has shown that *DUX4* although initially considered as a pseudogene, was expressed in FSHD but not control primary myoblasts [21]. DUX4 over-expression alters emerin distribution, and induces caspases 3/7 activation leading to death of established cell lines [22]. The DUX4 protein is a transcription factor that can activate or repress numerous genes. Its expression in C2C12 cells recapitulates key features of the FSHD molecular phenotype, including repression of MyoD and its target genes, diminished myogenic differentiation, repression of glutathione redox pathway components, and sensitivity to oxidative stress [23]. One additional DUX4 target is the paired-like homeodomain transcription factor 1 gene (*Pitx1*) on chromosome 5, that is specifically up-regulated in muscles of patients with FSHD as compared to 11 other neuromuscular disorders [21]. The PITX1 protein in turn activates genes involved in skeletal muscle atrophy that is a hallmark of FSHD [24]. Together these studies provided a link between the genetic defect at *D4Z4* (activating *DUX4* gene expression) and the pathophysiology of FSHD muscles and thus demonstrated a major role for DUX4 and PITX1 in the disease.

**Figure 1:**
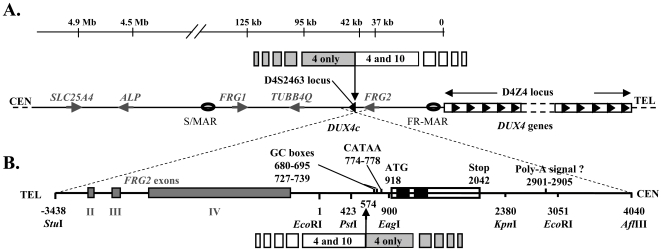
Localization of the *DUX4* and *DUX4c* genes. (A) Schematic representation of the 4q35 subtelomeric region with the *D4Z4* repeat array and the *SLC25A4* (previously known as *ANT1*) [41], *ALP* [60], *FRG1* [14], *TUBB4Q* [15] and *FRG2* [16] genes. *DUX4* maps within each *D4Z4* element [21] and *DUX4c* within an isolated inverted *D4Z4* unit at the *D4S2463* locus. S/MAR and FR-MAR: nuclear scaffold/matrix attachment regions, [13]. Upper line: 4q35/10q26 limit of homology [27]. (B) Enlargement (inverted orientation) of the 7.5-kb fragment that contains *DUX4c* with part of the *FRG2* gene. The *DUX4c* ORF is boxed, with the homeoboxes in black. The promoter GC boxes, the putative variant TATA box (CATAA) and polyadenylation signal are indicated. Numbering from the *Eco*RI site (GenBank acc. no. AY500824).

The evolutionary conservation of the *DUX4* ORF since placental mammals and the presence of several functional paralogues in rodents lend support to a defined function for double homeodomain proteins [25]. We have identified a human *DUX4* homologue that we named *DUX4c* (centromeric) mapping 42 kb proximal of the *D4Z4* array, next to the *FRG2* gene (Fig. 1A). *DUX4c* is inside a truncated and inverted solitary *D4Z4* unit [26] (locus *D4S2463*) mapped in 4q35 at the proximal limit of homology with 10q26 (Fig. 1A) [27].

A previous functional study indicated that the *DUX4c* protein could interfere with differentiation of mouse C2C12 cells [28]. We have now characterized the endogenous *DUX4c* gene located near the FSHD locus and shown it was functional. We could detect its expression in human muscle cells (biopsies and primary myoblasts) at RNA and protein levels, and found it was up-regulated in FSHD. DUX4c over-expression could activate human myoblast proliferation and inhibit their differentiation *in vitro*. This process is most probably caused through induction of the MYF5 myogenic regulator that is up-regulated by DUX4c.

## Results

### Characterization of the *DUX4c* gene

We have identified the *DUX4c* gene by analysis of a published genomic sequence (GenBank accession no. AF146191) containing *FRG1*, *TUBB4Q* and *FRG2* (Fig. 1A). Due to the high GC content, we have confirmed its sequence on two different genomic fragments (GenBank accession no. AY500824). *DUX4c* is identical on a large part of its coding and proximal sequences (including the two homeoboxes) to its *DUX4* homologue in *D4Z4*. The *DUX4c* ORF is located in a single exon and extends over 1,125 bp as compared to 1,275 for *DUX4*. Both genes are identical in a 1,137 bp fragment starting 111 bp 5′ from their common start codon, except for three mismatches outside the double homeoboxes.

### The *DUX4c* gene contains a functional promoter

**Figure 2:**
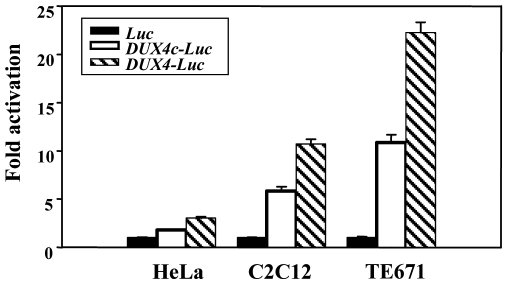
Transcriptional activity of the DUX4c gene. Transcriptional activity of the *DUX4c* promoter. HeLa, C_2_C_12_ and TE671 cells were transfected with *pGL3* vectors containing the luciferase reporter gene either promoterless (black bars) or fused to the *DUX4c* (white bars) or *DUX4 (*striped bars) promoter. Luciferase activity was measured 24 h post-transfection and expressed relative to the activity of the promoterless vector set to 1. The means and standard errors are indicated (n = 18).

A modified TATAA box (TACAA), and a GC box binding Sp1 mediate basal activity of the *DUX4* promoter [18]. *DUX4c* presents similar elements at slightly shifted positions, and an additional GC box (Supplemental Fig. S1). In order to evaluate the *DUX4c* promoter activity, we fused a 477-bp *Pst*I*/Eag*I upstream fragment to the luciferase reporter gene in *pGL3* and generated *pDUX4c-LUC* (Fig. 1B). The luciferase activity was assayed 24 h after transfection of C2C12 (mouse myoblast), TE671 (human rhabdomyosarcoma) or HeLa cells. As compared to the promoter-less vector (*Luc*), the luciferase activity was increased 6- and 11-fold in C2C12 and TE671 cells, respectively, but only 1.8-fold in HeLa cells (p<0.01) (Fig. 2).

### The DUX4c protein is up-regulated in FSHD primary myoblasts and biopsies

**Figure 3:**
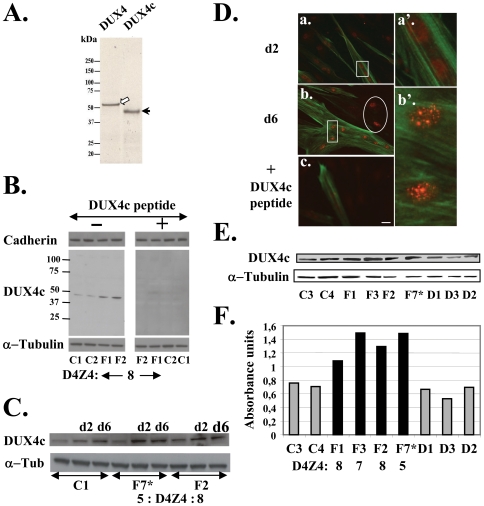
DUX4c protein expression in myoblasts. (A) Transcription/translation *in vitro* in a rabbit reticulocyte lysate in the presence T7 RNA polymerase and [^35^S]-cysteine with genomic fragments encoding DUX4 (lane 1) or DUX4c (lane 2) cloned in *pCIneo*. 52 kDa-DUX4 (white arrow) and 47 kDa-DUX4c (black arrow) are detected by autoradiography after 10% PAGE-SDS. (B–C) 30 µg total proteins extracted from primary myoblast were analysed by 4–12% PAGE-SDS and Western blot with the indicated primary antibodies, appropriate secondary antibodies coupled to HRP and the ECL kit. α-Tubulin was the loading control. (B) Competition: a 5-fold excess of DUX4c antigenic peptide was pre-incubated (+) or not (−) with the serum raised against DUX4c or cadherin as indicated. (C) Extracts were prepared either from proliferating myoblasts or 2 (d2) or 6 (d6) days after induction of differentiation. (D) DUX4c (red) was detected by immunofluorescence in nuclei of myoblasts and myotubes 2 (d2) and 6 (d6) days after inducing differentiation. a' and b' correspond respectively to two enlarged nuclei from d2 and d6 (white boxes in a and b). The labeling is weakened after competition with the immunogenic peptide (c). Troponin T (green) is a myotube differentiation marker. Myoblasts not fused to myotubes express DUX4c (red nuclei) but not troponin T. Bar corresponds to 20 µm. (E) 30 µg protein extracted from primary myotubes were analyzed by Western blot as in (C). (F) Densitometric scanning of the film shown in (E): DUX4c expression levels were normalized to α-Tubulin (relative absorbance units). C: control, F: FSHD, D: DMD.

Conceptual translation of the *DUX4c* gene yields a 374-residue protein identical to DUX4 (424 residues) in the double homeo- and most of the carboxyl-terminal domains (positions 1–342). The last 32 residues in DUX4c only present 40% identity with DUX4 (Supplemental Fig. S2). The DUX4 and DUX4c proteins presented apparent molecular weights (MW) of 52 and 47 kDa, respectively, in PAGE-SDS upon expression of their ORF by transcription-translation *in vitro* (Fig 3A). In order to investigate *DUX4c* expression in human muscle cells we raised a rabbit antiserum against a carboxyl-terminal peptide present in DUX4c but not in DUX4 (underlined in Supplemental Fig. S2).

This antiserum revealed a 47-kDa protein in Western blot of both control and FSHD primary myoblasts, but at a higher intensity in the later (Fig. 3B, left panel, lanes F1, F2). This band was specific since it disappeared upon competition with the DUX4c immunogenic peptide (Fig. 3B, right panel) or when the cells were transfected with a siRNA targeting the DUX4c 3′UTR (Supplemental Fig S3). During differentiation (2 and 6 days after confluence), the DUX4c protein progressively accumulated in both control and FSHD myoblasts (Fig. 3C).

This antiserum also detected DUX4c by immunofluorescence in the nuclei of primary myotubes, as expected for a homeodomain protein (Fig. 3D; a, enlarged in a'; 2-day differentiation). The punctuate labelling increased during differentiation (b, enlarged in b'; 6-day differentiation) and was lost upon competition with the DUX4c peptide (c). A myoblast subpopulation not included in myotubes also presented labelled nuclei (b, white circle).

We then compared DUX4c expression in control, FSHD or Duchenne muscular dystrophy (DMD). Since *DUX4c* was induced upon differentiation we performed Western blots on extracts of homogenous myotube populations (6-day differentiation) to quantify the protein (Fig 3E) relative to the α-tubulin loading control: a 1.5- to 2-fold increase was seen in FSHD as compared to control or DMD cells (Fig. 3F).

**Figure 4:**
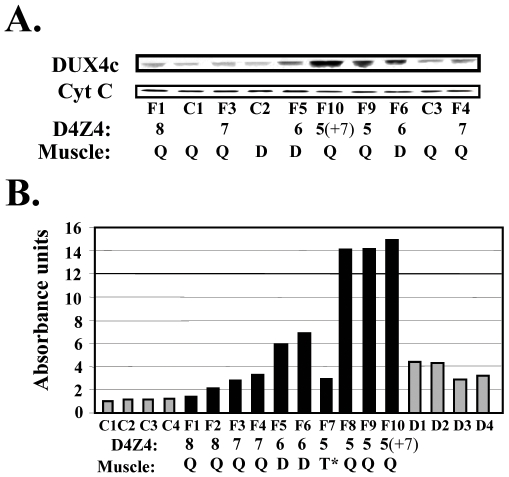
DUX4c protein expression in muscle biopsies. (A) 30 µg protein extracted of muscle biopsies were analyzed by Western blot as in Fig. 3, except that cytochrome C was the internal loading control. (B) Densitometric scanning of the Western blot shown in (A) and of additional samples (not shown): DUX4c expression levels were normalized to cytochrome C (relative absorbance units). Samples are indicated C1 to C4 for controls, F1 to F10 for FSHD, and D1 to D4 for DMD as well as the *D4Z4* copy numbers of the FSHD patients. The biopsied muscle is indicaded (D, Q: non-affected deltoid or *quadriceps*; T*: affected *trapezius*). F10 also has a D4Z4 array contraction on the second 4q35 allele (+7).

We similarly analysed DUX4c expression in proteins extracted from human muscle biopsies (Fig 4A.) The biopsies were taken in FSHD unaffected quadriceps (Q) or deltoid (D) except for one FSHD affected trapezius (T). Control biopsies were taken in the same muscles of non-affected individuals. The DUX4c signal was quantified relative to the cytochrome C loading control by densitometric analysis of this Western blot and of another one not shown (Fig. 4B). An increased DUX4c level was shown in all FSHD samples with the highest ones detected in the samples derived from patients with low *D4Z4* copy numbers (5 or 6; Fig. 4B), except for the F7 sample corresponding to the affected trapezius muscle that presented important necrosis and fat accumulation (data not shown). We observed a progressive DUX4c increase associated to decreasing *D4Z4* copy numbers. One of the samples was derived from a patient homozygous for the 4q35 deletion (5 and 7 *D4Z4* units) and presented a similar DUX4c level as those found in the biopsies derived from patients with 5 but not 7 *D4Z4* units. The nature of the 4q alleles (A or B) in this patient is unknown but only one allele might be pathogenic as reported for two other homozygous patients with FSHD [10]. DUX4c levels were also higher in DMD compared to control biopsies, in contrast to the levels observed in myoblast cultures.

### DUX4c over-expression induces MYF5 and cell proliferation

**Figure 5:**
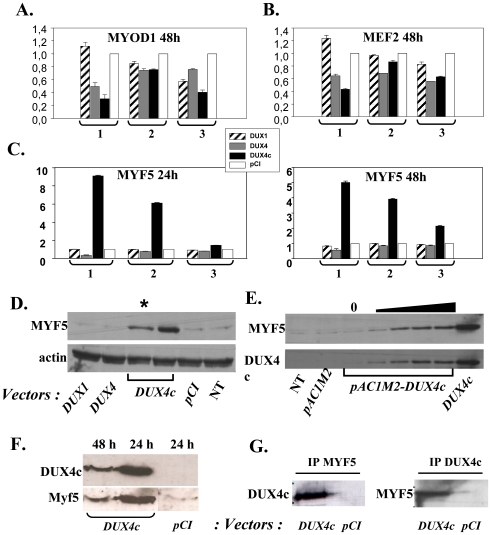
DUX4c over-expression induces MYF5. (A–C) TE671 cells were transfected with the indicated *pCIneo* vectors. Nuclear extracts were deposited in triplicate on a plate where the MYOD1, MEF2 or MYF5 specific DNA target was immobilized. The DNA-bound protein was detected by ELISA (TransAm assay). Relative absorbances are given relative to the insertless *pCIneo* sample arbitrarily set to 1. Three independent experiments (1 to 3) made in triplicate are presented. (D) TE671 nuclear extracts were prepared 48 h after transfection as above and 30 or 15 (*) µg were analyzed by 10% PAGE-SDS and Western blotting with a serum raised against MYF5 or actin (internal control). NT: non transfected cells. (E) same as in D but transfection was with *pAC1M2-DUX4c* and *DUX4c* expression induced by doxycycline (o to1000 ng). (F) Mouse C2C12 cells were transfected with the indicated *pCIneo* vectors. Total protein extracts were prepared 24 or 48 h later and 40 µg were analysed by Western blot with a serum raised against MYF5 or DUX4c as in D. (G) 40 µg nuclear extracts of TE cells transfected with the indicated vectors were subjected to immunoprecipitation with the anti-DUX4c or the anti-MYF5 serum. The immunoprecipitate was analysed by Western blot with the anti-DUX4c or anti-MYF5 serum as in D.

We investigated whether DUX4c might affect myogenic factor activities with ELISA-based assays that detect *trans*-factors upon binding to their immobilized DNA target (TransAm, Active Motif). We prepared nuclear extracts of human TE671 cells transfected with *pCIneo* vectors expressing DUX4c, DUX4 or the shorter DUX1 protein (a non-4q35 homologue limited to the homeodomains [29]) or with the insert-less vector. Both *DUX4c* and *DUX4* decreased MYOD1 at 24 h (not shown) and 48 h as well as the MEF2 family members evaluated at 48 h (Fig. 5A–B). *DUX4c* uniquely induced MYF5 binding activity at 24 and 48 h (Fig. 5C, p<0.001). A Western blot demonstrated that MYF5 was induced at the protein level and reached 5- to 6-fold 48 h after transfection (Fig. 5D). This induction was dose-dependent as shown in cells transfected with a doxycyclin-inducible DUX4c expression vector (Fig 5E).

Intriguingly in another study, the Myf5 mRNA was down-regulated in mouse C2C12 cells expressing an inducible DUX4c cassette [28]. To evaluate whether this discrepancy was caused by species differences we transfected C2C12 cells with *pCINeo-DUX4c*. Again the Myf5 protein was induced by DUX4c as compared to cells transfected with the insert-less *pCINeo* vector. Moreover, we could confirm the dose-dependence between DUX4c and Myf5 levels as observed in human muscle cells (Fig 5F).

We then evaluated whether the MYF5 protein might interact with DUX4c. TE671 cells were transfected with *pCINeo*-DUX4c and protein extracts prepared 48 h later. The immuno-precipitate obtained with an anti-MYF5 serum was loaded on a PAGE-SDS gel and a Western blot was performed with the anti DUX4c serum, showing the expected 47-kDa protein. The protein interaction was confirmed when the immuno-precipitaion was performed with the anti-DUX4c serum and the Western blot with the anti-MYF5 serum (Fig 5G).

**Figure 6:**
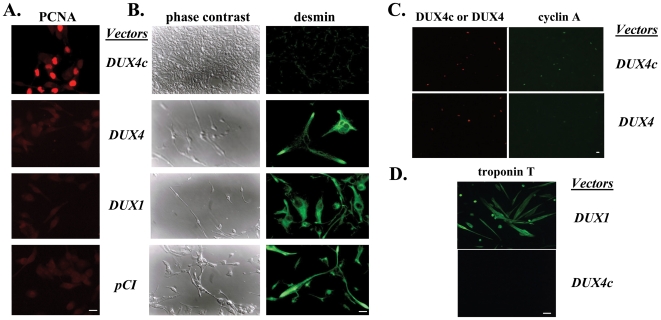
DUX4c over-expression induces cell proliferation. (A) PCNA was detected by immunofluorescence (red) 24 h post transfection with the *pCIneo* vectors indicated. The picture of DUX4c expressing cells was exposed 1.4 sec versus 2.7 sec for the other panels to visualize the cells. (B) The cells were switched 24 h post transfection to a differentiation medium, and observed 4 days later by phase contrast microscopy (left panels). Early differentiation was evaluated by desmin detection (green, right panels). (C) Cyclin A (green) and DUX4/4c (red) were detected by immunofluorescence 24 h post transfection of human immortalized myoblasts with the indicated *pCIneo*-vectors. (D) Human immortalized myoblasts were transfected with the indicated *pCIneo*-vectors and switched to differentiation medium 48 h later. Troponin T was detected by immunofluorescence 8 days later. Bars correspond to 20 µm.

As MYF5 expression and MYOD1 down regulation are related to the maintenance of the muscle satellite cell pool (reviewed by [30], [31]), we investigated whether DUX4c protein expression affected cell proliferation in human TE671 cells or immortalized primary myoblasts [32]. DUX4c induced a 2–3 fold higher proliferation rate than two controls (DUX1 or the insert-less *pCINeo* vector, p<0.005) as determined with a colorimetric MTT assay 24 and 48 h post transfection. The cells expressing DUX4 had a decreased proliferation rate as expected from its toxicicity [22]. These data were confirmed by total protein quantification and detection of two proliferation markers i.e. PCNA and cyclin A, in the DUX4c expressing cells (Fig. 6A,C).

We switched the different transfected TE671 cells to a low serum medium to induce differentiation. Four days later, all the cells showed cytoplasmic extensions and high immunofluorescence staining for desmin, an early myogenic differentiation marker, except for the DUX4c expressing cells. These were numerous and smaller and only had a weak labelling for desmin (Fig. 6B). Similarly, at a later time (8-day differentiation) troponin T, another marker of myoblast differentiation, was undetected in human immortalized myoblasts transfected with *pCI-neoDUX4c* while it was clearly induced in the other transfected cells (Fig 6D).

In conclusion, forced DUX4c expression induced MYF5 and cell proliferation suggesting a role in myoblast proliferation during muscle regeneration.

### Characterization of the endogenous *DUX4c* mRNAs

**Figure 7:**
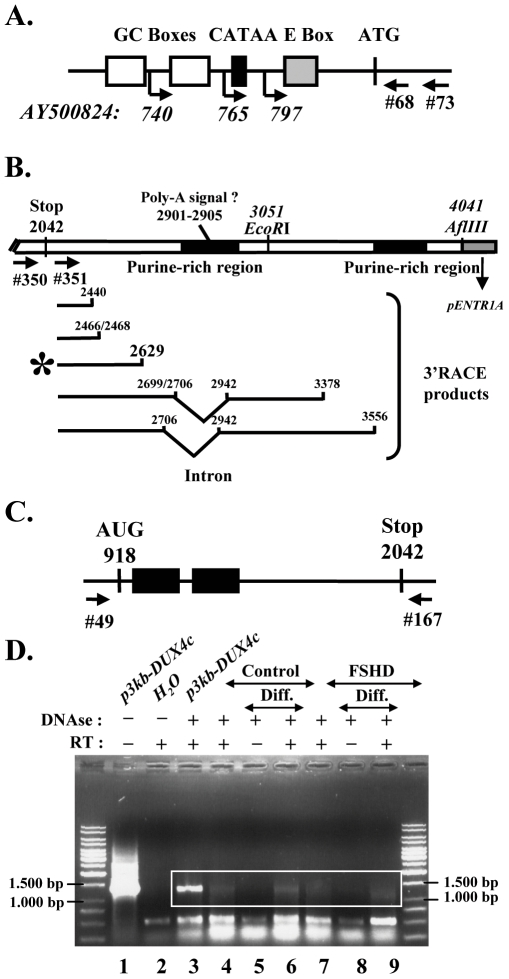
Characterization of the DUX4c mRNA. (A) Schematic representation of the *DUX4c* promoter with the transcription start sites (arrows and positions) identified by 5′ RACE (primer indicated) on RNA extracted from control and FSHD myoblasts. (B) Top: Schematic representation of the *p7.5 kb-DUX4c* insert (see Supporting Information S2) close to its 3′ cloning site, showing the stop codon, the putative poly-A addition signal, two purine-rich (86 and 83%) regions (black boxes) and the primers used in 3′RACE (arrows, #350 and 351). Bottom: Mapping of the multiple 3′ ends and alternative splicing detected in the 3′RACE products. These were derived from RNAs of either C2C12 cells transfected with *p7.5 kb-DUX4c* or FSHD primary myoblasts (*). (C) Schematic representation of the *DUX4c* ORF with the homeoboxes (black boxes) and the primers used for RT-PCR. (D) Amplification of the *DUX4c* mRNA was performed on total RNA extracted from FSHD (F24) or control primary myoblasts (C29) either in proliferation (lanes 4 and 7) or differentiated to myotubes (diff.). RNA samples were incubated (+) or not (−) with DNase I, and reverse transcriptase (RT) as indicated. The PCR products were analysed by electrophoresis on a 1%-agarose gel and stained with ethidium bromide. As a positive control (lane 3), RT-PCR was performed on RNA of C2C12 cells transfected with *p3 kb-DUX4c*.

In the last part of this study, we wanted to characterize the DUX4c mRNAs expressed in human myoblasts. Optimal 5′ and 3′ RACE conditions were established on mouse C2C12 cells transfected with the *DUX4c* genomic clones to avoid the background generated by hundreds of homologous human *DUX* genes [2], [29], [33]–[35] (see Supporting Information S1 and Supplemental Fig. S1). A 5′RACE performed on total RNAs of human primary myoblasts detected 2 and 1 initiation sites in control and FSHD cells, respectively, that might result from the use of either a GC or the variant TATAA box (Fig. 7A). A single DUX4c mRNA end (identical to the most frequent end observed in transfected mouse cells; position 2629) was detected by 3′RACE on total RNA of FSHD myoblasts (Fig 7B). The oligo-dT adapter used for the RT step of the 3′RACE suggested that the mRNAs were polyadenylated although no poly-A addition signal could be identified on the gene.

Since the DUX4c mRNA ends we mapped were flanking the complete ORF we selected primers to detect the full size mRNA by reverse transcription (RT) with a *DUX4c*-specific primer followed by PCR (Fig 7C, Supplemental Table S1). The RT-PCR was first performed on total RNAs of C2C12 cells transfected with the *DUX4c* genomic fragment and yielded the expected 1265-bp fragment (Fig. 7D, lane 3) that was missing upon RT omission or in cells transfected with the insertless vectors (data not shown). A sample not treated with DNase I provided a PCR positive control (lane 1). The 1265-bp RT-PCR product was detected at a very low intensity in a control and an FSHD myoblast line both in proliferation (lanes 4 and 7), and after differentiation to myotubes (lanes 6 and 9). It was absent upon RT omission (lanes 5 and 8). The RT-PCR products were cloned in *pCR4* and the *E. coli* colonies were screened by hybridization with a *DUX4c* specific probe (see Material and Methods), yielding 30–50% positives. Several of these clones were analyzed and their full cDNA sequences were found identical to *DUX4c* in either control or FSHD samples.

In aggregate, these data demonstrated that the *DUX4c* gene could be transcribed from its natural promoter into RNAs covering its entire ORF, and that such mRNAs were expressed in FSHD and control myoblasts.

## Discussion

In the present study, we have characterized the *DUX4c* gene mapped near the FSHD locus and have demonstrated it expressed a protein in human myoblasts and muscle biopsies.

Our demonstration of DUX4c mRNA expression confirmed that this 4q35 gene was in a chromatin structure compatible with transcription as suggested by previous studies: its promoter was found associated with acetylated histone H4 [36] and it interacted with RNA polymerase II at slightly higher level than non-transcribed sequences [37]. Moreover, *DUX4c* is brought in the same chromatin loop as the enhancer-containing *D4Z4* repeats in FSHD myoblasts [13], [17]; Fig. 1A) and this enhancer directly interacts with the DUX4c promoter [38]. However, these publications had failed to detect DUX4c mRNAs by RT-PCR. Our present study is the first report detecting the endogenous *DUX4c* mRNA and protein of human muscle cells. Similarly to the homologous *DUX4* mRNA, the *DUX4c* sequence is extremely GC-rich and difficult to retro-transcribe and amplify. We have recently listed the critical technical points to amplify these low-abundance and GC rich mRNAs, and highlighted the need for a higher temperature and a gene-specific primer during the RT step [21] (see Materials and Methods). An additional problem in the study by Alexiadis et al [37] is that the forward primer used to specifically amplify *DUX4c* hybridized at position 777–800, i.e. upstream of some of the 5′ ends found in the present study. Indeed, our 5′RACE data suggested that transcription could be driven as well by the variant TATAA box as by several GC boxes in the *DUX4c* promoter.

In the *DUX4c* gene, a single exon comprised the full ORF, and we found a spliced out intron in the 3′ untranslated region (UTR) of some mRNAs. No polyadenylation signal could be found on the gene and the *DUX4c* mRNAs presented heterogeneous 3′ ends. Such a structure was reported for histone genes that use the U7 snRNA to process the mRNA ends (37). However, the 3′RACE was done with an oligo-dT primer suggesting a polyadenylation of the DUX4c mRNAs.

The studies of gene transcription in 4q35 are complicated by the fact these genes belong to families with functional or pseudogene homologues in multiple locations of the human genome. No transcript could be detected for *TUBB4Q* [15], some studies found increased *FRG1* and *FRG2* mRNAs in FSHD samples [16], [39], others did not [36], [40]. The only protein expression data were reported for *DUX4* present within each D4Z4 repeated element [21] and for *ANT1* (also called *SLC25A4*) located 4.9 Mb from the locus. The ANT1 protein was strongly upregulated in contrast to its mRNA, suggesting a posttranscriptional regulation [36], [41].

Y. Vassetsky's group showed that an enhancer located in *D4Z4* interacts directly with the *DUX4c* promoter as a result of changes in chromatin looping caused by the *D4Z4* array contraction [38]. In agreement with this observation, we detected that the DUX4c protein was increased in extracts of FSHD muscles. Moreover, a progressive DUX4c accumulation was associated to decreasing *D4Z4* copy numbers, with DUX4c expression reaching 10-fold the control value when the pathogenic allele only presented 5 *D4Z4* units (F8–F10 Fig. 4B). A similar activation pattern was previously shown for the flanking *FRG2* gene that belongs to the same chromatin loop as *DUX4c* [39]. The DUX4c increase was observed in non-affected muscles of patients suggesting it was an early event in the disease progression. DUX4c expression could therefore be considered as a sensor of chromatin structure in FSHD. It would be very interesting to evaluate DUX4c expression in biopsies of the few reported patients with a *D4Z4* deletion removing *DUX4c* (see below) or of asymptomatic individuals with *D4Z4* deletion to confirm this hypothesis.

No link between DUX4c expression and *D4Z4* copy number could be observed in myoblasts. However the only primary myoblast line with 5 units we used derived from the F7 biopsy taken in an affected *trapezius*, not in a non-affected *quadriceps* like the other samples. The relationship between DUX4c expression and *D4Z4* copy number in muscle biopsies is in agreement with a transcriptional inhibitory role of the *D4Z4* element [39] and should be further evaluated in additional samples from different muscles and in patients with lower *D4Z4* copy numbers. We could not correlate DUX4c protein expression and residual D4Z4 copy number (5 to 8) to clinical disease severity in the present study. This is in agreement with previous data for which such a correlation could not be established for D4Z4 arrays larger than 3 units [7], [42]. The DUX4c protein has not been observed by other groups in proteome studies of FSHD muscles [41], [43] most probably because its very high pI (11.1) was not reached during the isoelectrofocalisation step.

Forced DUX4c expression in human muscle cells induced the MYF5 protein and its DNA-binding activity. This transcription factor is known to inhibit myoblast differentiation [44], [45]. Furthermore, DUX4c expression inhibited MYOD1 DNA-binding activity and prevented cell differentiation following serum withdrawal, as reported for Myf5^+^/MyoD^−^ myoblasts [31] and in iC2C12-DUX4c cells [28]. The later study reported a down-regulation of the Myf5 mRNA following DUX4c induction. In contrast, in the present report, we observed a dose-dependent induction of the Myf5 protein as well in human TE671 as in mouse C2C12 cells expressing DUX4c. In addition, we found an interaction between DUX4c and Myf5 that might lead to a stabilisation of the later protein. Indeed Myf5 degradation is known to be controlled by specific posttranslational modifications [46] and it is therefore possible that deregulation of the mRNA does not lead to change in the protein levels.

Besides an interference with myoblast differentiation that was also described by Bosnakovski et al (2008), we found that DUX4c over-expression in human cells led to an increased proliferation rate by MTT assay, PCNA and cyclin A labelling. This phenomenon could be due to MYF5 protein accumulation since its absence was shown to reduce the proliferation rate of satellite cell derived myoblasts [47], [48]. The DUX4c increase in DMD muscle biopsies (Fig. 4A–B) could be related to the higher regeneration rate reported for this disease [49]. The DMD biopsies used in the present study indeed contained newly formed fibres still presenting centrally located nuclei (data not shown). The DUX4c up-regulation in the DMD biopsies (Fig. 4B) could be related to the increased satellite cell proliferation in comparison to control muscles where satellite cells were quiescent. In keeping with this idea, no difference in DUX4c expression was found between DMD and control myoblasts that were both derived from activated satellite cells (Fig 3E). In contrast to FSHD, DMD muscles do not present D4Z4 contraction (see Supplemental Table S2) therefore DUX4c up-regulation could only be related to increased muscle regeneration.

The up-regulation of DUX4c expression upon myoblast differentiation and its inhibitory effect on this very process appear contradictory. However one could hypothesize that DUX4c has different function in myoblasts or in myotubes according to its interaction with different protein partners. Indeed its MYF5 partner which is involved in proliferation is only expressed in myoblasts but not in myotubes [50]. Moreover the observed change of DUX4c nuclear localization during differentiation is in favour of a functional switch.

Functional studies performed in parallel on the homologous DUX4c and DUX4 proteins have shown several differences despite their high similarity. Both proteins share an identical double homeodomain and specifically bind the *Pitx1* promoter but DUX4 activates its transcription at a stronger level [21]. DUX4 does indeed present a carboxyl terminal region partly missing in DUX4c that mediates strong transcriptional activation [51], [52]. Forced DUX4c expression in TE671 cells did not induce caspases 3/7 activity nor cell death as shown for DUX4 [22] (A. Marcowycz, unpublished data). Moreover, we found that the MYF5 induction was unique to DUX4c expression.

The *DUX4c* gene is only present on chromosome 4 where it defines the proximal limit of homology with chromosome 10, and our results suggest it plays a role in FSHD that is uniquely associated with array contractions in 4q35 [8]. However, a deletion extending from the *D4Z4* repeat array to include *FRG2* and *DUX4c* was reported in some families with FSHD [53] suggesting that neither gene could cause the disease. A transvection effect resulting from a misbalance of chromatin and transcription factors at 4q35 and unrelated loci following the deletion [12] was proposed to explain that the *FRG2* mRNA expressed in FSHD myoblasts originated mostly from the homologous *FRG2* gene on chromosome 10, not 4 [16]. A similar mechanism in *trans* might also occur between the two chromosome 4 alleles in FSHD cells, activating *DUX4c* on the non-affected one. This could be tested in muscle biopsies of patients with an extended 4q35 deletion removing *DUX4c* on one allele. Nevertheless, in most affected families, both *DUX4c* and *FRG2* are present and could contribute to the penetrance and severity of the disease. Alternatively, since we found DUX4c expression in control myoblasts and muscle biopsies, it is possible that a single allele deletion could also have pathological consequences.

We have demonstrated functionality of the *DUX4c* gene in spite of a variant TATAA box, an intron-less ORF and the lack of a poly-A addition signal. If the other 3.3-kb repeated elements scattered on other chromosomes could similarly be expressed, the human genome might have to be expanded with hundreds of additional *DUX* genes. We have previously characterized other actively transcribed *DUX* genes on the acrocentric chromosomes [33]. One *DUX* gene with introns was proposed to have generated multiple retrotransposed pseudogenes with reported EST on autosomal chromosomes [35]. Four putative *DUX* genes were reported in the pericentromeric region of the Y chromosome [34], [54]. Together with our protein expression data on *DUX4* and *DUX1* [21], [29] this result bears on the general questions of how to define a gene versus a pseudogene, and of what can be considered as junk or “func” (functional) DNA [55], [56].

FSHD is a complex disease associated with a chromatin change affecting the expression of several genes. However to date only two proteins (i.e. ANT1 and DUX4) were shown to be up-regulated from FSHD candidate genes. Although *DUX4* activation strikingly recapitulates key features of the FSHD molecular phenotype [21], [23], other 4q35 genes could also contribute to the heterogeneity of the FSHD phenotype [57]. This could be the case for FRG1 that is implicated in muscle development and angiogenesis [19], [58] but at the present time no data on FRG1 protein expression in FSHD muscle is available. The present study demonstrated that the DUX4c protein is over-expressed in FSHD muscle and could therefore contribute to the development of the disease. Moreover, we have uncovered a putative role for DUX4c in muscle regeneration that should be further investigated in injured or atrophic muscles of healthy individuals, and in muscles of patients with different neuro-muscular pathologies.

## Materials and Methods

### Ethics Statement

Muscle biopsies (see Supplemental Table S2) were performed according to a procedure approved either by the University of Rochester Research Subjects Review Board (reference number RSRB#8567) or current ethical and legislative rules of France as described [41] (ref number 050503). Written informed consent was obtained from all subjects, as directed by the ethical committee of either institution. In addition, the uses of this material have been approved by the ethics committee of the University of Mons-Hainaut (ref number A901).

### Mammalian Cell Cultures

C2C12 and TE671 cells were grown in DMEM, 1% penicillin/streptomycin/fungizone (Cambrex, Verviers, Belgium) and 10% fetal calf serum (PAA Laboratories) at 37°C under 5% CO_2_. HeLa cells were grown in DMEM-F12 (Cambrex) supplemented as above. The primary myoblast cultures were established as described [41] and grown in DMEM with 10% FCS and 1% Ultroser G (BioSepra, Cergy-Pontoise, France). For differentiation, the cells were either grown to confluence or the medium was replaced by DMEM supplemented by 2% horse serum (PAA laboratories, Pasching, Austria) as indicated. Immortalized myoblasts were grown and differentiated as reported (Zhu et al) except that 1% Ultroser G was used instead of HGF during proliferation.

### Plasmid constructs

A 477-bp *Pst*I/*Eag*I fragment corresponding to the *DUX4c* promoter was fused to the luciferase reporter gene in *pGL3* (Promega, Leiden, The Netherlands) yielding *pGL3-DUX4c*. The *pGL3control* has the SV40 promoter/enhancer (Promega). A 3-kb *Eco*RI fragment containing the DUX4c natural gene was cloned in *pENTR1A* (Invitrogen, Carlsbad, CA), yielding *p3 kb-DUX4c*. The 1.2-kb *DUX4c* ORF was cloned into *pCIneo* (Promega) or *pAC1M2* (for doxycyclin induction [59] yielding *pCIneo-DUX4c* or *pAC1M2-DUX4c*. All the constructs were confirmed by sequence determination and are detailed in Supporting Information S2.

### Transient luciferase expression

Either 10^5^ C2C12, 2×10^5^ TE671 or 4×10^5^ HeLa cells were seeded in each well of 6-well plates and grown overnight. Transfections were performed with 1.6 µg reporter plasmid and 16 ng *phRL-SV40* (internal control) per well with either FuGENE6 (Roche Diagnostics, Mannheim, Germany) for TE671 cells or Lipofectamin2000 (Invitrogen) for C2C12 and HeLa cells. Cells were lysed 24 h later with the dual luciferase assay system (Promega) and activity measured on a Packard LumiCount (PerkinElmer). The firefly luciferase reporter plasmids were derived from *pGL3* (Promega) and contained either no insert (*pGL3-Basic*), the *DUX4c* or *DUX4* promoter [18]. Experiments were done 3 times in triplicate with 2 different preparations for each plasmid (n = 18 for each point). The *DUX4c* promoter was about 40- and 350-fold less active in muscle and HeLa cells, respectively, than the SV40 promoter/enhancer *(pGL3-Control*, not shown).

### RT-PCR, 5′ and 3′ RACE

Total RNA was extracted and DNase-treated as described previously [21]. RT was done on 2.5 µg freshly prepared RNA with primer # 167 (all the primers sequences are given in Supplemental Table S1) and 200 U of SuperScript III with a procedure for high secondary structure [21]. 8 µl cDNA were used for PCR with primers # 49 and # 167 and the conditions were 3 min at 94°C, followed by 1 min at 94°C, 1 min at 68°C with a 1°C decrease at each cycle, and 2 min at 68°C for 4 cycles, followed by 31 cycles of 1 min at 94°C, 1 min at 64°C, 2 min at 68°C with 5 sec/cycle increment during elongation. The RT step of the 5′ and 3′ RACE was performed with 10 or 2 µg of total DNase-treated RNA, respectively, with the RLM-RACE kit (Ambion, Austin, TX). *DUX4c* primers #68 and #73 (5′RACE) or #350 and #351 (3′RACE) were used for the nested PCR. The products were cloned in *pCR4* (TOPO TA kit, Invitrogen), amplified in *E. coli* and sequenced with the CEQ 2000 (Beckman Coulter).

### Antibodies against DUX4c

A rabbit antiserum was raised against a 16-residue peptide (underlined in Supplemental Fig. S2) specific of the DUX4c carboxyl-terminal domain. This peptide was chosen by accessibility prediction programs, synthesized, coupled to KLH and injected into rabbits. The resulting antisera were purified by affinity chromatography on the immobilized peptide (Eurogentec, Seraing, Belgium).

### Transcription/translation *in vitro*

Aliquots of rabbit reticulocyte lysate (TNT kit, Promega) were incubated with *pCIneo* vectors in the presence of 30 µCi [^35^S]-cysteine (Amersham Biosciences, Roosendaal, The Netherlands) and T7 RNA polymerase. 10 µl of the products were denatured in XT sample buffer with reducing agent (Bio-Rad, Hercules, CA) and analysed by PAGE-SDS. The gel was incubated 30 min in Amplify (Amersham Biosciences), air dried and submitted to autoradiography.

### Western blot

Whole cell extracts of myoblast primary cultures were obtained by lysis in 50 mM Tris pH 7.0, 50 mM NaCl, 0.1% Nonidet P40, 1 mM DTT and protease inhibitors, were separated by PAGE-SDS and electrotransferred onto a PVDF or nitrocellulose membrane according to the manufacturer (Amersham Biosciences). The Western blot was incubated with the rabbit anti-DUX4c (1∶1000) or anti-Myf5 (1∶500, C-20, Santa Cruz Biotechnology, Santa Cruz, CA) sera followed by secondary antibodies coupled to HRP and the ECL kit (Amersham Biosciences).

For standardization, the membranes were stripped and immunostaining was performed with primary antibodies raised against either α-tubulin (mAb, Sigma-Aldrich, Saint Louis, MO), pan-cadherins (rabbit serum, Sigma-Aldrich), cytochrome C (rabbit serum, Santa Cruz Biotechnology) or actin (rabbit serum, Sigma-Aldrich) as indicated.

### Co-immunoprecipitation

1.5×10^6^ TE671 cells were seeded in a 75-cm^2^ flask, grown and transfected with *pCIneo* plasmids. Whole cell extracts were prepared 24 h later using sonication in 500 µl lysis buffer follow by centrifugation 5 min at 16.000 g to discard cell membranes. Immunoprecipitation was performed on 800 µg total extract with the mouse monoclonal 9A12 antibody directed against DUX4 and cross-reacting with DUX4c (1∶5, Dixit 2007) or rabbit anti-MYF5 (1∶100, Santa Cruz Biotechnologies) serum in 1 ml IP buffer in the presence of protein G-agarose (Fermentas) or protein A-Sepharose (Amersham Biosciences) respectively as described by the manufacturers. The final pellet was heated 5 min at 95°C in 30 µl loading buffer with reducing agent (Fermentas), and centrifuged 5 min at 16,000 g. The supernatant was analysed by 12% PAGE-SDS followed by electrotransfer to a PVDF membrane as above, and Western blot was performed with the anti-MYF5 serum (1∶500) or the 9A12 antibody (1∶1000) followed by secondary antibodies coupled to HRP (Amersham Biosciences) and revealed as above.

### Immunofluorescence

1.5×10^5^ TE671 cells were seeded on coverslips in 6-well plates and transfected 24 h later with 1 µg plasmid DNA as indicated. After 24 h, the cells were fixed in 4% paraformaldehyde or Carnoy (desmin detection). Immunostaining was performed by standard procedures as detailed in Supporting Information S2 with anti-DUX4c (1∶50) or anti-PCNA (PC10, 1∶40, Dako, Glostrup, Denmark) serum or anti-desmin (DE-R-11, 1∶50, Dako) or anti-cyclin A (1∶50, BD Transduction Laboratories, Erembodegem, Belgium) antibodies. As a control, the anti-DUX4c serum was preincubated 2 h with a 5-fold molar excess of the DUX4c immunogenic peptide. The anti-IgG secondary antibodies were either coupled to FITC or biotinylated (Dako, Amersham Biosciences) and incubated with streptavidin-Texas-Red (Vector Laboratories, Burlingame, CA). The primary myoblasts (on collagen-coated dishes) were incubated with anti-DUX4c and anti-troponin T (1∶100, JLT-12, Sigma) followed by Alexa secondary antibodies (goat anti-mouse 488 and anti-rabbit 555, Invitrogen).

### Proliferation assay

1.5×10^5^ TE671 cells were seeded in 6-well plates, grown overnight and transfected with 1 µg plasmid DNA. The CellTiter 96 non-radioactive cell proliferation assay (Promega) was used 24 or 48 hours after transfection as described by the manufacturer. Experiments were done in triplicate.

### Myogenic factor activities

1.2×10^6^ TE671 cells were seeded in a 75-cm^2^ flask, grown overnight, transfected with 10 µg of either *pCIneo-DUX* plasmid and collected 48 h later. The cell lysates were prepared as described in Supporting Information S2 and were deposited on an ELISA plate where a specific DNA target was immobilized (TransAm kit, ActiveMotif, Carlsbad, CA). A specific rabbit antiserum was added, followed by a secondary antibody coupled to HRP, and a substrate yielding a product with absorbance at 450 nm.

### Statistical analyses

Statistical significance was evaluated by the Student *t* test.

## Supporting Information

Supporting Information S1 Supplemental data
(0.05 MB DOC)

Supporting Information S2 Supporting Materials and Methods
(0.04 MB DOC)

Table S1 Primer sequences
(0.06 MB DOC)

Table S2 Biopsies and myoblast lines.
(0.05 MB DOC)

Figure S1 Characterization of DUX4c mRNA in transfected cells. Alignment of the DUX4c and DUX4 promoter sequences (GenBank accession nos AY500824 and AF117653). The numberings start at the 5′EcoRI sites. The variant TATAA boxes are underlined with brackets, the putative E boxes double underlined, the GC boxes are boxed, and the translation initiation codons circled. The broken arrows indicate the transcription start sites experimentally determined for DUX4 (CoppÃ©e et al, 2004) and DUX4c. The later ones were identified by 5′RACE on RNA extracted from C2C12 cells transfected with p3 kb-DUX4c (a) and p7.5 kb-DUX4c (b). At each start site, the consensus initiator sequences is shown in low cases (c/t c a n t/a c/t c/t). The primers (dotted line) used in a chromatin immunoprecipitation study of acetylated histone H4 in 4q35 (Jiang et al, 2003) map in DUX4c.
(1.38 MB TIF)

Figure S2 Sequence alignment of the DUX4c and DUX4 proteins. The DUX4c protein sequence was derived from pSK-DUX4c (integrating variations mentioned in GenBank accession no. AY500824) and the DUX4 protein from GenBank #AF117653. The identical double homeodomains are boxed. The arrows indicate polymorphic residues: either valine or isoleucine at position 229 in both DUX4c and DUX4; either alanine or proline at position 272 in DUX4c, but only proline in DUX4. The peptide used to generate a specific rabbit antiserum against DUX4c is underlined.
(2.56 MB TIF)

Figure S3 Downregulation of DUX4c expression by a RNA silencing. Human muscle TE671 cells were transfected (siPORT NeoFX, Ambion) or not (-) with 20 nmol of siRNA either targeting the DUX4c 3′UTR, an unrelated genomic sequence (unrl), or a sequence not found in the human genome (negative control, n.c.) (Ambion). They were either transfected 5 h later (Fugene 6) with the pCIneo-DUX4c expression vector (DUX4c) or not (NT). Protein extracts were prepared 72 h later and analysed by Western blot with the rabbit anti-DUX4c antiserum as in Fig 3. Actin (antibody from Sigma) was used as a loading control.
(2.71 MB TIF)
