# Supplementary material for: DUX4c Is Up-Regulated in FSHD. It Induces the MYF5 Protein and Human Myoblast Proliferation
Source: PLoS One. 2009 Oct 15;4(10):e7482. doi: 10.1371/journal.pone.0007482 (PMC2759506; doi:10.1371/journal.pone.0007482)
Supplement: Supporting Information S1 — Supplemental data (0.05 MB DOC) [file pone.0007482.s001.doc]

**Supplemental data**

**Characterization of the *DUX4c* gene.**

We have identified the intron-less *DUX4c* gene by analysis of a published genomic sequence (GenBank accession no. AF146191) containing *FRG2*. Because these regions have a very high GC content, we wanted to confirm the *DUX4c* sequence on two different genomic clones. The first one was a 2.4-kb *Eco*RI/*Kpn*I subclone of the c34 cosmid [1] that we named *p2.4kb-DUX4c.* Its sequence was identical to the #AF146191 reverse/complement except for a C→T transition at coordinate 1,421. In addition, we subcloned a 3-kb *Eco*RI fragment from *PAC 202J3*, yielding *p3kb-DUX4c*: we determined its sequence GenBank accession no. AY500824) that presented the same transition and 5 additional mismatches with #AF146191. All these differences involved a G or a C, and two were missense polymorphisms (see protein study below).

We aligned the *DUX4c* sequence with the homologous *DUX4* gene that we had previously identified in the two 3.3-kb elements left in the *D4Z4* locus of a patient ([2]; GenBank accession no. AF117653). The 3-kb *Eco*RI fragment containing *DUX4c* presented about 80% identity with the 3.3-kb *Kpn*I fragment containing *DUX4*. A putative variant poly-adenylation signal (GATAAA instead of AATAAA; [3]) was found 859 bp downstream from the *DUX4c* stop codon.

**Characterization of the *DUX4c* mRNA ends.**

We first set up the experimental conditions on mouse C2C12 cells transfected with the *DUX4c* genomic clones (*p3kb-DUX4c*, *p7.5kb-DUX4c*) or *pCIneo-DUX4c* in which the *CMV* enhancer/promoter is coupled to the *DUX4c* ORF (positive control). Total RNAs were extracted 24h post-transfection and submitted to RNA ligase-mediated rapid amplification of 5’ ends (5’RACE) using a method that only targets capped mRNAs. For the positive control (transfection with *pCIneo-DUX4c*) the RT and nested PCR (primers # 68 and # 73, see Table 1) yielded the 600-bp fragment expected for transcription from the *CMV* promoter. 5’RACE products of about 110, 300 (highest abundance) and 450 bp were obtained from cells transfected with the *DUX4c* natural gene (not shown). These were lacking in transfections with the insert-less vectors or when the tobacco acid pyrophosphatase (TAP) that removes the mRNA 5’ cap was omitted. The products were cloned and individual sequence start sites (a,b) were reported in Fig S1A. Besides one that was within the ORF (not shown) the 9 sites mapped over a 200-bp region around two GC boxes and the CATAA sequence, and fitted with the consensus initiator sequence (C/T CAN T/A C/T C/T; [4]). The 300-bp 5’RACE product started at coordinate 873, just 3’of a putative GC box conserved in *DUX4*. Another mapped 3’ of an E box shared with *DUX4* and where a start site had been found.

We then performed 3’RACE with a nested PCR (primers #350 and #351; Fig. S1B) on total RNA of C2C12 cells transfected as above. A major product was found with the *DUX4c* natural gene but lacking in the negative controls (transfections with the insert-less vectors, not shown). The 3’RACE products were cloned in bulk and sequenced. Multiple ends were detected following cloning of the PCR products and sequencing (from position 2440 to position 3556, major product position 2629:* in Fig. 7B). The larger fragments had one intron spliced out that removed the putative variant polyadenylation signal therefore not functional (Fig. 7B). This putative variant polyadenylation signal was not found in any RT product because either they were too short, or it mapped within the spliced intron. There was no A stretch just 3’ of the different ends mapped on the *DUX4c* gene that could allow hybridization of the oligo dT adapter used for the RT step, suggesting that the mRNAs were polyadenylated post transcription. In replication-dependent histone genes that lack such signal, a conserved hairpin structure in the 3’ UTR and a specific interaction of a purine-rich region with the U7 snRNA allow processing of the mRNA ends [5]. The *DUX4c* 3’ UTR harbours 2 purine-rich regions (>80%), several UUUC motifs and multiple direct and inverted repeats predicted to form hairpins (GeneBee; [6]) that might be similarly used.

**Characterization of the DUX4c protein.**

The *DUX4c* gene encodes a putative 374-residue protein with a theoretical molecular weight of 39.4 kDa. Its sequence was aligned with the homologous DUX4 protein (Figure S2) showing that the double homeodomain and most of the carboxyl-terminal domain (residues 1-342) were identical. DUX4c was 50-residue shorter and its last 32 amino acids only presented 40 % identity with DUX4. The nucleotide sequence differences described above in *DUX4c* genomic fragments only resulted in two amino acid changes. The Val/Ile 229 polymorphism was observed both in DUX4c and DUX4; while the Ala/Pro 272 polymorphism seems specific to DUX4c since published *DUX4* sequences only encode Pro272. The theoretical isoelectric point of DUX4c was 11.1 (www.expasy.org), i.e. much higher than for DUX4 (pI: 8.7).

Three other amino acid changes (L11F, P316S and V365L) were deduced from polymorphisms of genomic DUX4c sequences reported on Genome Browser website UCSC ([http://genome.ucsc.edu/](http://genome.ucsc.edu/index.html) : Human Mar. 2006 (hg18) assembly) [7,8].

**Bibliography**

1. Hewitt JE, Lyle R, Clark LN, Valleley EM, Wright TJ et al. (1994) Analysis of the tandem repeat locus D4Z4 associated with facioscapulohumeral muscular dystrophy. Hum Mol Genet 3: 1287-1295.

2. Gabriels J, Beckers MC, Ding H, De Vriese A, Plaisance S et al. (1999) Nucleotide sequence of the partially deleted D4Z4 locus in a patient with FSHD identifies a putative gene within each 3.3 kb element. Gene 236: 25-32.

3. Wickens M, Stephenson P (1984) Role of the conserved AAUAAA sequence: four AAUAAA point mutants prevent messenger RNA 3' end formation. Science 226: 1045-1051.

4. Carey M, Smale TS (2004) A Primer on Transcriptional Regulation in Mammalian Cells. In: Transcriptional Regulation in Eukaryotes:Concepts, Strategies, and Techniques. New York: Cold Spring Harbor Laboratory Press. pp. 1-50.

5. Zanier K, Luyten I, Crombie C, Muller B, Schumperli D et al. (2002) Structure of the histone mRNA hairpin required for cell cycle regulation of histone gene expression. RNA 8: 29-46.

6. Brodsky L.I., Ivanov V.V., Kalai dzidis Ya.L., Leontovich A.M., Nikolaev V.K. et al. (1995) GeneBee-NET:Internet-based server for analyzing biopolymers structure. Biochemistry 60: 923-928.

7. Karolchik D, Baertsch R, Diekhans M, Furey TS, Hinrichs A et al. (2003) The UCSC Genome Browser Database. Nucleic Acids Research 31: 51-54.

8. Kent WJ, Sugnet CW, Furey TS, Roskin KM, Pringle TH et al. (2002) The human genome browser at UCSC. Genome Research 12: 996-1006.
