# Supplementary material for: DUX4c Is Up-Regulated in FSHD. It Induces the MYF5 Protein and Human Myoblast Proliferation
Source: PLoS One. 2009 Oct 15;4(10):e7482. doi: 10.1371/journal.pone.0007482 (PMC2759506; doi:10.1371/journal.pone.0007482)
Supplement: Supporting Information S2 — Supporting Materials and Methods (0.04 MB DOC) [file pone.0007482.s002.doc]

**Supporting Materials and Methods**

**Plasmid constructs.** A 2.4-kb *Eco*RI/*Kpn*I genomic subcloned in the *pSK(+)* plasmid (Stratagene, La Jolla, CA) corresponding to the *D4S2463* locus and derived from the c34 cosmid [1] was kindly provided by Dr. J. Hewitt (University of Nottingham, UK): it contains the *DUX4c* gene and was renamed *p2.4kb-DUX4c*. A 477-bp *Pst*I/*Eag*I fragment corresponding to the *DUX4c* promoter was fused to the luciferase reporter gene in *pGL3* (Promega) yielding *pGL3-DUX4c*. The *pGL3control* has the SV40 promoter/enhancer (Promega). The 202J3 PAC isolated from the RPCI-6 library (detailed on www.lgtc.nl) and kindly provided by Dr. S. van der Maarel (University of Leiden, The Netherlands) contained a genomic fragment extending from the *D4S2463* to the *D4Z4* loci. *PAC* DNA was digested either with *Eco*RI or with *Stu*I and *Afl*III (converted to blunt ends) for subcloning in *pENTR1A* (Invitrogen), at either the *Eco*RI or *Sma*I site, yielding vectors that contain the *DUX4c* gene either alone (*p3kb-DUX4c*) or with the *FRG2* structural part excluding exon I (*p7.5kb-DUX4c*).

The 1.2-kb *DUX4c* ORF was amplified by PCR from *p2.4kb-DUX4c* with the *Pfu* polymerase (Promega), in the presence of 0.4 M sulfolane (Acros Organics, Gell, Belgium) according to a method for GC-rich DNA [2] with primers # 49 and 167 or # 345 and 353 (see Table 1) that contained an *Eco*RI and an *Xba*I site or an *Sal*I and *Not*I site, respectively. The fragment was subcloned into *pCIneo* (Promega) or *pAC1M2* [3] yielding *pCIneo-DUX4c* or *pAC1M2-DUX4c*.

All the constructs were confirmed by sequence determination. For transfections plasmids were endotoxin-free (Ultra-Mobius kit, Merck Biosciences, Darmstadt, Germany).

**Sequence determinations and analyses.** The sequences were determined using the CEQ DTCS kit and a Beckman CEQ 2000 XL DNA analysis system (Beckman Coulter, Fullerton, CA). The primers used (see Table 1) were either from Invitrogen or Eurogentec. Computer programmes were used through the Belgian node of the European Molecular Biology Organization (http://www.be.embnet.org). The *p3kb-DUX4c* sequence was deposited in Genbank (AY500824) and differed from the published AF146191 sequence in 6 positions.

**Immunofluorescence.** After fixation, TE671 cells were treated with PBS-Triton 0.2 % (PBS-T) and blocked in normal goat serum in PBS-T with 50 mM NH_4_Cl at 25°C. The cells were incubated with the primary antibody followed by washing in PBS-T and incubation with the appropriate secondary antibody as mentioned in the text. For the primary myoblast, cells were rinsed with PBS-Triton 0.5% (PBS-T), blocked in PBS-BSA 0.5 %, incubated with the primary antibody followed by washing in PBS- and incubation with the appropriate secondary antibody as mentioned in the text. Observations were made under a fluorescence microscope or a phase contrast microscope.

**Myogenic factor activities.**

The nuclear extracts were made as follows (all the steps are performed at 4°c): the cells were rinced once with PBS, once with PBS/Na_2_MoO_4_(1 mM)/NaF(5 mM), and incubated for 3 min in hypotonic buffer pH 7,4 (20 mM Hepes, 5mM NaF, 1mM Na_2_MoO_4_, 0,1 mM EDTA). The cells were lysed in 1ml of lysis buffer (hypotonic buffer + 0,5% NP40) on ice and scrapped. The lysate was then centrifuged for 30 sec at 16000g. The pellet was resuspended in 50 µl of resuspension buffer, constituted of 5 % glycerol, proteases inhibitors, 4% phosphatase inhibitor cocktail (25 mM NaVO_3_, 250 mM 4-nitrophenyl phosphate, 250 mM β-glycerophosphate, 125 mM NaF). Then 50 µl of saline buffer (5% glycerol, 0,4 M NaCl, proteases inhibitors, 4% phosphatase cocktail inhibitor) were added. The lysate was incubated for 30 min under rotation and centrifuged for 10 min at 16000 g. The protein concentrations were determined in the supernatant by the Bradford assay (Bio-Rad protein assay, Bio-Rad).

in 50 µl of 5 % glycerol, proteases inhibitors, 4% phosphatase inhibitor cocktail (25 mM NaVO3, 250 mM 4-nitrophenyl phosphate, 250 mM β-glycerophosphate, 125 mM NaF).

Five µg were deposited on an ELISA plate where a specific DNA target was immobilized (TransAm kit, ActiveMotif).

**Bibliography**

1. Hewitt JE, Lyle R, Clark LN, Valleley EM, Wright TJ et al. (1994) Analysis of the tandem repeat locus D4Z4 associated with facioscapulohumeral muscular dystrophy. Hum Mol Genet 3: 1287-1295.

2. Chakrabarti R, Schutt CE (2001) The enhancement of PCR amplification by low molecular-weight sulfones. Gene 274: 293-298.

3. Chtarto A, Yang X, Bockstael O, Melas C, Blum D et al. (2007) Controlled delivery of glial cell line-derived neurotrophic factor by a single tetracycline-inducible AAV vector. Experimental Neurology 204: 387-399.
