# Supplementary material for: DUX4c Is Up-Regulated in FSHD. It Induces the MYF5 Protein and Human Myoblast Proliferation
Source: PLoS One. 2009 Oct 15;4(10):e7482. doi: 10.1371/journal.pone.0007482 (PMC2759506; doi:10.1371/journal.pone.0007482)
Supplement: Table S1 — Primer sequences (0.06 MB DOC) [file pone.0007482.s003.doc]

# Table S1: primer sequences

| # | Orient. | Primer sequence | Position in #**AY500824** |
| --- | --- | --- | --- |
| **145** | F | 5’ GTGTCTCCCGCCCCCGAAAG 3’ | 214-233 |
| **110** | F | 5’ ACCCACCCACCCCACAAG 3’ | 682-699 |
| **49** | F | 5’ cggaattcggttctggttctggttCGCGTCCGTCCGTGAAATTCC 3’ | 880-900 (10791-10811 in #**AF117653**) |
| **345** | F | 5' gtcgacgccaccATGGCCCTCCCGACACCCT 3' | 918-936 |
| **57** | F | 5’ cggaattcggttctggttctggtAGACGCGGCCCGCCAGAAG 3’ | 1179-1197 |
| **32** | F | 5’ atgGAATCGAAGGGCCAGGCAC 3’ | 1346-1364 |
| **20** | F | 5’ CTTTCGTGAGCCAGGCAG 3’ | 1534-1551 |
| **85** | F | 5’ GCTTGCGCCACCCACGTCCCAG 3’ | 1811-1832 |
| **134** | F | 5’ GCGGCAAGCACAGATGCC 3’ | 1941-1958 |
| **350** | F | 5’ ACAGTCACCTCCAGCCTGTTAT 3’ | 2007-2028 |
| **351** | F | 5’ AACCTCTGACTCGGTATGGAGA 3’ | 2234-2255 |
| **137** | F | 5’ CCCAGCGTTCTTCAGTCGAG 3’ | 2476-2494 |
| **138** | R | 5’ GTCCATCCTTCCGTCTGC 3’ | 199-182 |
| **73** | R | 5’ AGCTGGCGTGACCTCTCATTCTGA 3’ | 1141-1118 |
| **70** | R | 5’ ttctgcagGGGCCAGGGCCGAGATTC 3’ | 1175-1158 |
| **68** | R | 5’ TGCCTGGCCCTTCGATTCTGAAAC 3’ | 1363-1340 |
| **58** | R | 5’ ttctgcagGGCGACCCACGAGGGAGCAG 3’ | 1454-1435 |
| **86** | R | 5’ CTGGGACGTGGGTGGCGCAAGC 3’ | 1832-1811 |
| **184** | R | 5’ ATAACAGGCTGGAGGTGACTGTAG 3’ | 2028-2005 |
| **353** | R | 5' atagtttagcggccgCTGTGTCTACAGGAGCTCAT 3' | 2029-2048 |
| **165** | R | 5’ gcggatccCCACGTCCTTCAGCTCCC 3’ | 2112 2095 |
| **167** | R | 5’ gctctagaCCACGTCCTTCAGCTCCC 3’ | 2112-2095 |
| **111** | R | 5’ CCCCAACCCCGCTCCAAC 3’ | 2195-2178 |
| **146** | R | 5’ AGTCAGAGGTTCACCGCGAAAG 3’ | 2244-2223 |
| **136** | R | 5’ GGTCAGCTGGAAGGGAAGGC 3’ | 2280-2261 |
| **90** | F | 5’ atggaattcggttctggttctggtaGTCACCCTGCTCCCTCGT 3’ | 11340-11357 (#**AF117653**) |
| **118** | R | 5’ ttgaattcaTCACCGGGCCTAGACCTAGAA 3’ | 12262-12242 (#**AF117653**) |
| **273** | F | 5’ TGAATAGGGTCAGGGTGCTC 3’ | 118473-118493 (#**AF146191**) |
| **274** | R | 5’ CCCAGGTGCAGACCTTGTAT 3’ | 118672-118653 (#**AF146191**) |
| **193** | F | 5’ GCAGGCTTTAAAGGAACCAA 3’ | 451-470 (pENTR1A*) |
| **194** | R | 5’ ACTTTGTACAAGAAAGCTGGGTCT 3’ | 967-944 (pENTR1A*) |
| **T7** | F | 5’ TAATACGACTCACTATAGGG 3’ | 626-645 (#**X52328**) |
| **T3** | R | 5’ TTAATTGGGAGTGATTTCCC 3’ | 791-772 (#**X52328**) |

The orientation is given, either forward (F) or reverse (R). Primers were used as described in Materials and Methods and/or for sequence determinations. Additional sequences (smaller cases) of primers #57, #70, #58 and #165 were of no use in this study. The *pENTR1A sequence is available on www.lifetech.com. The sequences referred in GenBank are the following: #AY500824 (*DUX4c*), #AF117653 (*DUX4*), #146191 (a genomic 4q35 fragment) and #X52328 (pBluescript II SK(+) vector).
