# Supplementary material for: DUX4c Is Up-Regulated in FSHD. It Induces the MYF5 Protein and Human Myoblast Proliferation
Source: PLoS One. 2009 Oct 15;4(10):e7482. doi: 10.1371/journal.pone.0007482 (PMC2759506; doi:10.1371/journal.pone.0007482)
Supplement: Table S2 — Biopsies and myoblast lines. (0.05 MB DOC) [file pone.0007482.s004.doc]

**Table S2: Biopsies and myoblast lines.**

| # | Biopsy | Muscle | Myoblast  Line | Sex | Age | Disease | *D4Z4* units | *Analyses* |
| --- | --- | --- | --- | --- | --- | --- | --- | --- |
| C1 | x | Q | X | M | 39 | C | > | W.B. ; 5' and 3'R |
| C2 | x | D | X | F | 17 | C | > | W.B. |
| C3 | x | Q | X | M | 24 | C | > | W.B. |
| C4 | x | Q | X | M | 35 | C | > | W.B. |
| C5 |  | Q | X | M | 43 | C | > | I.F. |
| C29 |  | Q | X | M | 23 | C | > | RT-PCR |
| D1 | x | P | X | M | 12 | D | > | W.B. |
| D2 | x | P | X | M | 14 | D | > | W.B. |
| D3 | x | P | X | M | 15 | D | > | W.B. |
| D4 | x | K | X | M | 9 | D | > | W.B. |
| F1 | x | Q | X | F | 43 | F | 8 | W.B. |
| F2 | x | Q | X | F | 21 | F | 8 | W.B. |
| F3 | x | Q | X | M | 38 | F | 7 | W.B.; 5' and 3'R |
| F4 | x | Q |  | F | 39 | F | 7 | W.B. |
| F5 | x | Q | X | F | 20 | F | 6 | W.B. |
| F6 | x | D |  | F | 21 | F | 6 | W.B. |
| F7 | x | T* | X | M | 31 | F | 5 | W.B. |
| F8 | x | Q |  | F | 51 | F | 5 | W.B. |
| F9 | x | Q |  | M | 51 | F | 5 | W.B. |
| F10 | x | Q |  | F | 53 | F | 7 + 5 | W.B. |
| F24 |  | Q | X | M | 41 | F | 3 | RT-PCR |

C : control ; D : Duchenne muscular dystrophy ; F : FSHD ; * affected muscle

Q : quadriceps ; D : deltoid ; T : trapezius ; P : paravertebral ; K : knee ; S : sub-scapularis

W.B. : Western blot ; 5' and 3'R : 5' and 3'RACE ; I.F. : immunofluorescence.
