# Supplementary figures and images for: DUX4c Is Up-Regulated in FSHD. It Induces the MYF5 Protein and Human Myoblast Proliferation
Source: PLoS One. 2009 Oct 15;4(10):e7482. doi: 10.1371/journal.pone.0007482 (PMC2759506; doi:10.1371/journal.pone.0007482)

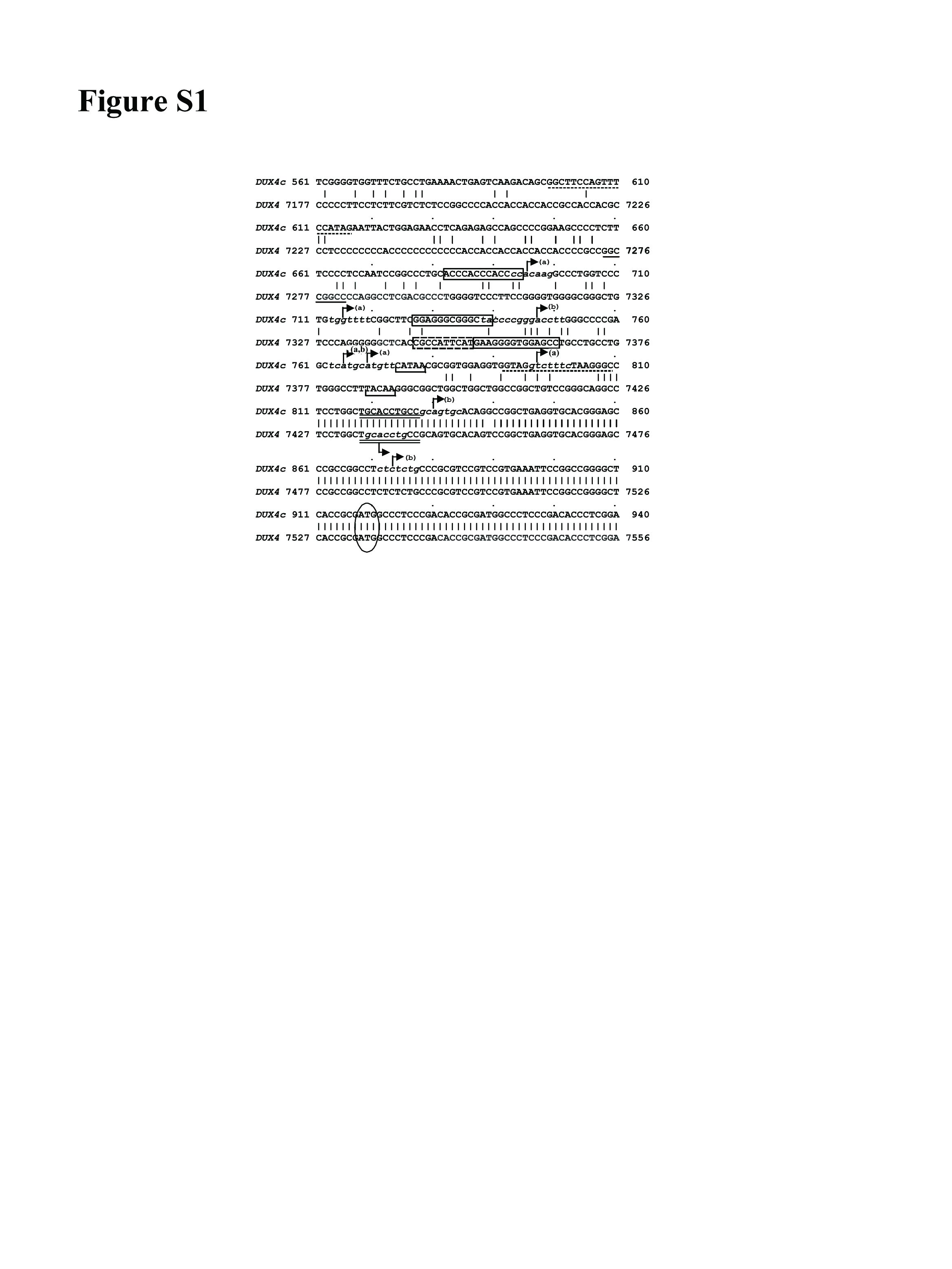

Supplement: Figure S1 — Characterization of DUX4c mRNA in transfected cells. Alignment of the DUX4c and DUX4 promoter sequences (GenBank accession nos AY500824 and AF117653). The numberings start at the 5′EcoRI sites. The variant TATAA boxes are underlined with brackets, the putative E boxes double underlined, the GC boxes are boxed, and the translation initiation codons circled. The broken arrows indicate the transcription start sites experimentally determined for DUX4 (CoppÃ©e et al, 2004) and DUX4c. The later ones were identified by 5′RACE on RNA extracted from C2C12 cells transfected with p3 kb-DUX4c (a) and p7.5 kb-DUX4c (b). At each start site, the consensus initiator sequences is shown in low cases (c/t c a n t/a c/t c/t). The primers (dotted line) used in a chromatin immunoprecipitation study of acetylated histone H4 in 4q35 (Jiang et al, 2003) map in DUX4c. (1.38 MB TIF) [file pone.0007482.s005.tif]

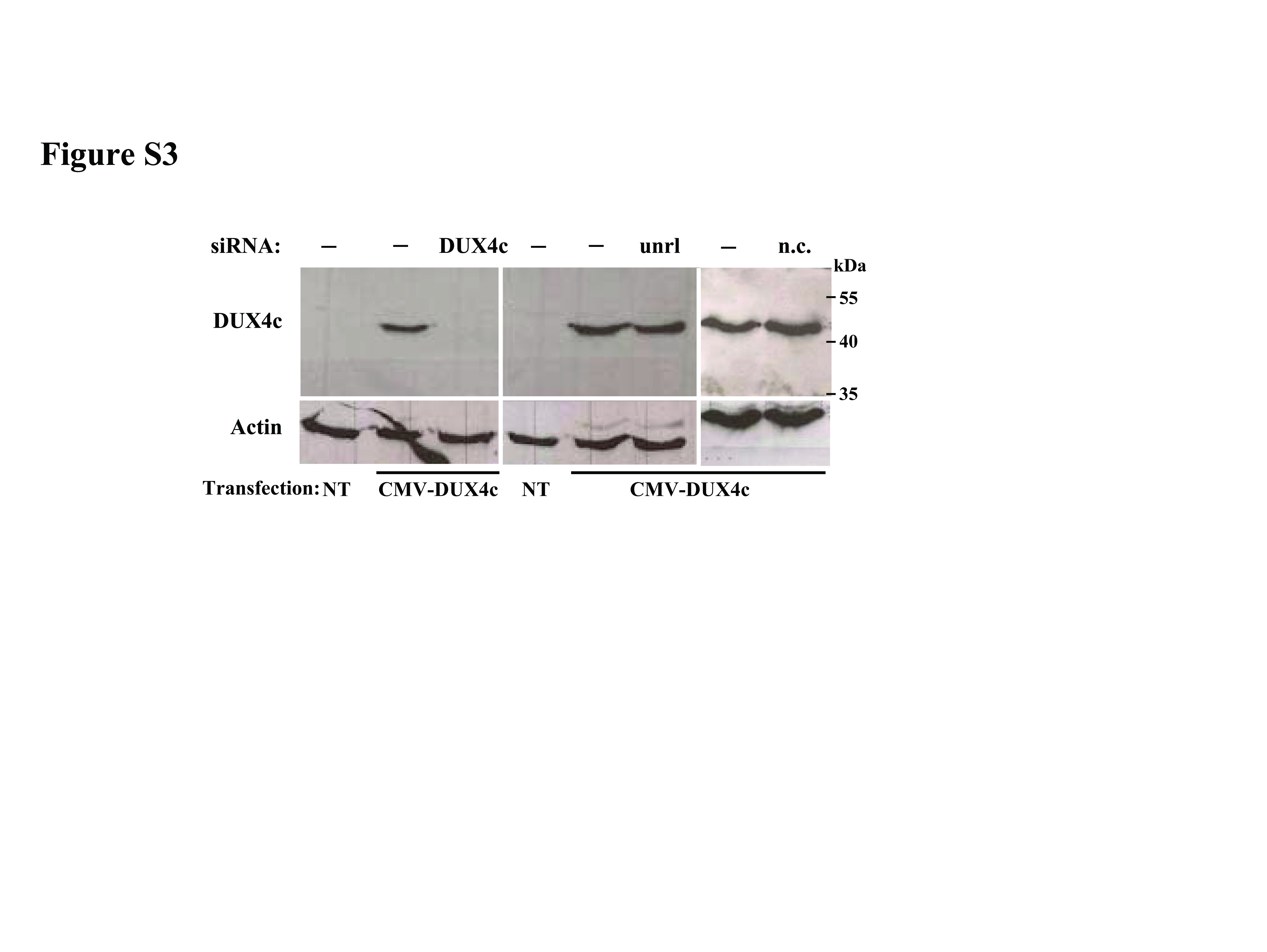

Supplement: Figure S3 — Downregulation of DUX4c expression by a RNA silencing. Human muscle TE671 cells were transfected (siPORT NeoFX, Ambion) or not (-) with 20 nmol of siRNA either targeting the DUX4c 3′UTR, an unrelated genomic sequence (unrl), or a sequence not found in the human genome (negative control, n.c.) (Ambion). They were either transfected 5 h later (Fugene 6) with the pCIneo-DUX4c expression vector (DUX4c) or not (NT). Protein extracts were prepared 72 h later and analysed by Western blot with the rabbit anti-DUX4c antiserum as in Fig 3. Actin (antibody from Sigma) was used as a loading control. (2.71 MB TIF) [file pone.0007482.s007.tif]
